# Modeling hypertrophic cardiomyopathy with human cardiomyocytes derived from induced pluripotent stem cells

**DOI:** 10.1186/s13287-022-02905-0

**Published:** 2022-06-03

**Authors:** Jiangtao Li, Xin Feng, Xiang Wei

**Affiliations:** 1 Division of Cardiothoracic and Vascular Surgery, Tongji Hospital, Tongji Medical College, Huazhong University of Science and Technology, No. 1095 Jiefang Avenue, Wuhan, 430030 Hubei China; 2 Division of Cardiothoracic and Vascular Surgery, Tongji Hospital, Tongji Medical College, Huazhong University of Science and Technology, Wuhan, Hubei, China; Key Laboratory of Organ Transplantation, Ministry of Education; NHC Key Laboratory of Organ Transplantation; Key Laboratory of Organ Transplantation, Chinese Academy of Medical Sciences, No. 1095 Jiefang Avenue, Wuhan, 430030 Hubei China

**Keywords:** Hypertrophic cardiomyopathy, Induced pluripotent stem cells, iPSC-derived cardiomyocytes, Disease modeling

## Abstract

One of the obstacles in studying the pathogenesis of hypertrophic cardiomyopathy (HCM) is the poor availability of myocardial tissue samples at the early stages of disease development. This has been addressed by the advent of induced pluripotent stem cells (iPSCs), which allow us to differentiate patient-derived iPSCs into cardiomyocytes (iPSC-CMs) in vitro. In this review, we summarize different approaches to establishing iPSC models and the application of genome editing techniques in iPSC. Because iPSC-CMs cultured at the present stage are immature in structure and function, researchers have attempted several methods to mature iPSC-CMs, such as prolonged culture duration, and mechanical and electrical stimulation. Currently, many researchers have established iPSC-CM models of HCM and employed diverse methods for performing measurements of cellular morphology, contractility, electrophysiological property, calcium handling, mitochondrial function, and metabolism. Here, we review published results in humans to date within the growing field of iPSC-CM models of HCM. Although there is no unified consensus, preliminary results suggest that this approach to modeling disease would provide important insights into our understanding of HCM pathogenesis and facilitate drug development and safety testing.

## Introduction

Hypertrophic cardiomyopathy (HCM) is a fatal heterogeneous myocardial disease, caused by autosomal dominant sarcomeric gene mutations, which manifests as left ventricular hypertrophy, myocardial hypercontractility, diastolic dysfunction, myofibrillar disarray, and fibrosis [1–3]. Epidemiological studies based on echocardiography have shown a prevalence of 1/500 in the population [1, 4], but a higher prevalence (about 1/200) when clinical and genetic diagnoses (including family members) are taken into account [5, 6]. Pare et al. [7] reported that mutation at the protein level was found in the MYH7 gene, encoding the *β*-myosin heavy chain (*β*-MHC). Based on this study, Seidman’s team identified missense mutations in the MYH7 gene that were associated with the first HCM [8]. Subsequently, numerous genetic studies have shown that HCM is an inherited disease of the cardiac sarcomere. Furthermore, the advent of next-generation sequencing and whole-exome sequencing led to the discovery of new HCM mutations in sarcomeric and sarcomere-associated genes [9, 10], reconfirming that HCM is primarily a monogenic sarcomeric disease.

Approximately half of HCM patients harbor mutations in genes that encode sarcomeric proteins and related myofilament elements responsible for regulating cardiomyocyte contraction and cardiac function [11–13]. Among the known causal genes, MYH7 and myosin-binding protein C (MYBPC3) are the most common, and they are responsible for about half of familial HCM patients [11, 14, 15]. Mutations of TNNT2, TNNI3, and TPM1 are relatively uncommon, together accounting for less than 10% [16–18]. Although less common, cardiac *α*-actin (ACTC1), myosin light chain 2 (MYL2), myosin light chain 3 (MYL3), and cysteine- and glycine-rich protein in 3 (CSRP3) have also been identified as causes of HCM [19–21]. The nine genes mentioned above have the strongest causal role in HCM (Table 1) [9, 22].

**Table 1:** Function and population frequency of common HCM causal genes

Gene	Protein	Function	Population frequency (%)
MYH7	*β*-Myosin heavy chain	ATPase activity and force generation	~ 15
MYBPC3	Myosin-binding protein C	Regulator of myocardial contraction and relaxation	~ 20
TNNT2	Cardiac troponin T	Regulator of actin–myosin interaction	~ 2
TNNI3	Cardiac troponin I	Inhibitor of actin–myosin interaction	~ 2
TPM1	*α*-tropomyosin	Places the troponin complex on cardiac actin	~ 2
ACTC1	Cardiac *α*-actin	Major constituent of contractile apparatus	< 1
MYL2	Regulatory myosin light chain	Affects actin–myosin dissociation and regulates contraction	~ 1
MYL3	Essential myosin light chain	Binds to myosin heavy chain and stabilizes myosin conformation	< 1
CSRP3	Cysteine- and glycine-rich protein 3	a Z disk protein; establishment and maintenance of the cytoskeleton	< 1

For common HCM mutations, such as MYH7 and MYBPC3, their causal role is unambiguous. However, not all HCM mutations cause HCM [23]. Even in the same family, HCM mutations typically show different expressivity (defined as the severity of the phenotype that develops in patients with the pathogenic mutation) and penetrance (defined as the proportion of individuals carrying a pathogenic mutation who display a phenotype) [24]. Because of human genetic diversity, population-specific frequency of variants, and the presence of thousands of coding variants in each exome, it is difficult to distinguish whether causal or incidental variants cause HCM [23, 25–28].

Although we have identified many mutations that cause HCM, our understanding of the pathogenesis from mutation to phenotype remains incomplete. The reason for this lies not only in the diversity of mutations that lead to similar clinical manifestations but also in the fact that animal models only partially recapitulate human phenotypes. For example, in mouse models, heterozygous mutations in MYH7 or MYBPC3 failed to develop pathognomonic septal hypertrophy seen in patients [29]. Neither has left ventricular obstruction been observed in any mouse models. Either homozygous knock-in or knock-out of the respective gene is lethal (e.g., *α*-MHC [30]), or animals develop severe left ventricular dysfunction (e.g., MYBPC3 [31–38]). The mouse reports still did not answer some fundamental questions: (1) the exact physiological roles of the most common sarcomeric proteins in HCM such as *β*-MHC and cMyBPC; (2) the mechanisms by which these sarcomeric proteins ensure normal cardiac systolic and diastolic function; and (3) the mechanisms of myocardial hypertrophy and disarray in HCM due to mutations in sarcomeric and non-sarcomeric genes with a different function [15, 29, 39, 40].

In addition, another reason for our limited understanding of the pathophysiology of HCM is that few myocardial tissues and cells have been isolated from HCM patients. And only a very small number of studies have specifically reported on the in vitro phenotype of HCM [41]. The myocardial tissues are commonly obtained from HCM patients undergoing surgical septal myectomy or heart transplantation. However, all these tissue sources represent an advanced stage of HCM, making us wonder to which extent the abnormalities in comparison with the heart tissue without heart failure can reflect primary defects or secondary compensatory outcomes.

The somatic cell reprogramming technique discovered by Yamanaka [42] paved the way for the generation of cardiomyocytes derived from patient-specific induced pluripotent stem cells (iPSCs), which allowed us to deeply understand the pathogenesis of HCM. The primary cause of HCM is the mutation in sarcomeric genes, leading to changes in Ca^2+^ handling properties, ion channel remodeling, energy deficiency, and microvascular dysfunction. However, the mechanisms why progressive changes initiated by these primary mutations occur in one individual and not in others are still unknown. Therefore, iPSC models represent a valuable tool to study HCM in vitro. By improving protocols for the generation of iPSC lines and the differentiation of cardiomyocytes from iPSCs, researchers can recapitulate in vitro phenotypes of HCM. iPSC-derived cardiomyocytes (iPSC-CMs) have several advantages: (1) They can be generated from a wide variety of readily available cells, including those in the skin, urine, and blood [43–45]; (2) they resemble the early stage of cardiac development and have the ability to show morphological and functional changes that first appear in disease without being hidden by systemic compensatory responses [46]; and (3) they are patient-specific, enabling genotype–phenotype association and providing an unprecedented platform for the drug screening for individualized therapy.

Up to date, much progress has been made utilizing patient-specific iPSC-CM models to characterize HCM and study the pathogenesis of HCM. In this review, we will provide an in-depth overview of current iPSC-CM models of HCM, including the generation and functional parameters of iPSC-CMs, methods of maturing iPSC-CMs, gene editing in iPSCs, and the HCM phenotype of iPSC-CMs. Finally, we will discuss the future perspectives of iPSC-CM models of HCM.

## Generation of iPSC-CM models

### iPSC generation

As a new approach to mimicking human diseases and associated genetic mutations, iPSC technology became possible since Yamanaka identified four critical reprogramming factors (OCT4, SOX2, KLF4, c-Myc). Depending on the type of donor cells, the combination of reprogramming factors might vary with the fact that specific cell types may endogenously express some factors. For example, c-Myc is not necessary for the reprogramming of fibroblasts [47]. Patient-specific iPSC-CM models require obtaining human tissues to generate iPSC lines. To minimize the invasiveness, a gradual shift from using fibroblasts from skin biopsies to urine or blood cells can be observed [44, 45].

Initially, iPSCs were generated by retroviral transduction [42, 48, 49]. Yamanaka's laboratory used the Moloney-based retroviral vector system, which generated iPSCs with high efficiency, but was restricted to dividing cells. Therefore, lentiviruses were used to improve the transduction efficiency of both dividing and non-dividing cells. However, it was found that the expression of Yamanaka factors was difficult to silence after lentiviral transduction [50, 51], resulting in difficulty in the differentiation of iPSCs [52]. Therefore, inducible systems were used to silence reprogramming factors so that they were not expressed [53]. However, the integration of viruses into host cell DNA increases the risk of insertional mutations [54]. For example, transgenic reactivation of c-Myc showed increased tumorigenicity limiting their application [47].

To overcome the disadvantages of retroviruses and lentiviruses, non-integrating vectors have been developed, including adenovirus, Sendai virus, virus-free methods like episomal transfection, and synthetic mRNA delivery. In host cells, adenovirus transduction allows the overexpression of reprogramming factors without genomic integration [55]. Sendai virus [56] is an RNA virus that does not enter the nucleus, thus reducing the risk of genomic insertion. Episomal transfection [57] is an alternative method to generate virus-free iPSCs. However, the efficiency of this approach is relatively low [58]. In addition, transfection using synthetic mRNA [59] is a simple, non-integrating strategy with high efficiency, which can overcome innate antiviral responses. Non-integrating vectors generate iPSCs that are more suitable for disease modeling.

In addition to integrating and non-integrating vectors, there are transgene-free reprogramming methods that can also generate iPSCs by implanting recombinant reprogramming factors into somatic cells [60]. Studies have shown that the reprogramming efficiency was improved by using small molecule compounds, e.g., the histone deacetylase inhibitor valproic acid [61].

In summary, different integration vectors, non-integration vectors, and non-transgenic reprogramming methods have been developed to improve efficiency and further reduce the risk of genome alteration.

### iPSC-CM differentiation

The adult heart is a post-mitotic organ with a very limited regeneration capacity [62]. However, in most cases, it is difficult to isolate primary human cardiomyocytes from surgical specimens and culture them for a long time due to ethical and technical issues. In addition, cardiomyocytes isolated from animals have species differences, e.g., different electrophysiology in comparison with human ones. Therefore, iPSCs are an important source of cardiomyocytes [63].

The differentiation of cardiomyocytes from human iPSCs can be achieved under in vitro culture conditions by modulating the signaling pathways involved in cardiac development [64]. Currently, there have been three main strategies for the directed differentiation of cardiomyocytes from iPSCs: co-culture with visceral endoderm-like (END-2) cells [65], embryoid body (EB)-based differentiation [64], and monolayer cell culture [66].

During embryonic development, END-2 cells release the factors that lead to cardiac differentiation around the mesoderm [67]. This finding was the basis of the co-culture strategy in which iPSCs can differentiate into cardiomyocytes with the presence of END-2 cells. Although this culture protocol successfully directed the differentiation of iPSCs into cardiomyocytes, the yield was very low (less than 10%) [68]. EB-based differentiation is a serum-mediated three-dimensional (3D) culture approach that relies on the ability of iPSCs to form floating cell aggregates in the low-adherent matrix. These aggregates called EBs can differentiate into cells of all three germ layers. However, due to the presence of serum, this method has low reproducibility and large inter-group differences [69]. Therefore, serum was then replaced by cytokines and growth factors, such as Wnt proteins [70], bone morphogenetic proteins (BMPs), and activin A [71, 72]. In addition, some small molecules could promote cardiac differentiation, including activators (CHIR99021) and inhibitors (IWR, XAV, IWP2) of the Wnt pathway [73]. However, by using this approach, the number of cells required is large and the differentiation efficiency is low. To overcome these disadvantages, the protocol of monolayer cell culture was developed [74, 75]. This approach can significantly increase the differentiation efficiency of iPSCs with derived cells exhibiting characteristic phenotypes of ventricular, atrial, or junctional cardiomyocytes [76]. Although the details, efficiency, and yield vary widely in various studies, the monolayer cell culture protocol has been used by an increasing number of investigators, probably because of its simplicity and high efficiency [77, 78].

### iPSCs and genome editing

iPSC technology has unprecedented advantages. However, using healthy relative- or healthy unrelated donor-derived iPSC-CMs as standard controls in early studies was not adequate [79]. This is because the phenotype differences between patient-derived iPSC-CMs and controls may be the result of different (epi)genetic backgrounds rather than disease-specific variants, or the effect of (epi)genetic backgrounds on phenotype may outweigh that caused by mutations. For example, the action potential duration of different iPSC-CM lines varies largely in healthy controls [80]. Furthermore, it has been reported that the monozygotic twins carrying the same MYH7 mutation differed significantly in the degree of myocardial fibrosis [81]. Therefore, it is sometimes difficult to link direct effects on the function with specific mutations by using healthy controls, as the (epi)genetic background of these cells is largely unknown [82].

Genome editing techniques like CRISPR-Cas9 [83], CPF1 [84], and TALENs [85] enable the generation of isogenic cell lines, which differ only in the mutation being studied and retain the same (epi)genetic background. Thus, the effects of mutations can be directly compared with their isogenic wild-type controls. Among all genome editing techniques, CRISPR-Cas9 is the first choice due to its low cost, simple structure, and high fidelity. Gene editing techniques can make precise changes to the genome, ranging from insertions or deletions (such as the ~ 65 kb Dip2a gene [86]) to the point mutation of a single base [87]. By combining iPSCs with genome editing techniques, it is possible to directly compare iPSC-CMs carrying disease-associated mutations with their corresponding isogenic wild-type controls to determine the exact effect of the mutation on disease [88].

Gene editing technologies are based on endonuclease activity, which aims to insert double-strand breaks (DSBs) into the genome at a precise and desired site. There are two main mechanisms for the repair of DSBs: nonhomologous end joining (NHEJ) and homology-directed repair (HDR) [89]. NHEJ is an efficient but error-prone process that may cause insertion, deletion, or substitution of nucleotides [90]. Insertion/deletion frequently results in frameshift mutations, which lead to premature termination codons (PTCs) [11]. HDR is a more accurate method for the specific repair of DSBs. Based on the use of a homologous template (single-stranded oligonucleotides or double-stranded DNA templates), HDR allows the precise introduction of point mutation [88], micro-peptide-encoding tags [91], and even fluorescent proteins at specific positions [92–94]. Thus, HDR can be targeted either to introduce specific mutations in healthy iPSCs or to correct preexisting genetic mutations to generate isogenic lines. However, the ratio of HDR is lower compared to NHEJ, limiting the efficiency of gene knock-in or introducing specific mutations [95]. Therefore, different approaches have been developed to inhibit NHEJ or promote HDR [96, 97]. For example, covalent linkage of DNA repair template to CRISPR-Cas9 nuclease can improve HDR efficiency, which may be a promising strategy [98, 99]. In addition, an alternative is using base editors that replace bases in the target DNA without breaking double-stranded DNA and DNA repair templates [100].

## iPSC-CMs characterization

It is indispensable that iPSC-CMs recapitulate the phenotypes of adult cardiomyocytes for disease modeling. However, iPSC-CMs generated using standard protocols differ from adult cardiomyocytes in cellular morphology, sarcomeric protein isoforms, electrophysiology, excitation–contraction coupling, and calcium handling, as well as mitochondrial function and metabolic characteristics. Table 2 summarizes the major phenotypic differences between iPSC-CMs and adult cardiomyocytes.

**Table 2:** Major differences between human iPSC-CMs and adult cardiomyocytes

	iPSC-CMs	Adult cardiomyocytes
Cellular morphology	Smaller in size, roundish in shape	Larger in size, elongated in shape
	Disorganized sarcomeres	Organized sarcomeres
Sarcomeric protein isoforms	Slow skeletal troponin *I* (ssTnI)	Cardiac troponin *I* (cTnI)
	Higher ratios of *α*-MHC/*β*-MHC	Lower ratios of *α*-MHC/*β*-MHC
	Titin N2BA isoform	Titin N2B isoform
Electrophysiology	Smaller maximum diastolic potential	Larger maximum diastolic potential
	Slower upstroke velocity	Faster upstroke velocity
	Automaticity in ventricular-like iPSC-CMs	No automaticity in adult ventricular myocytes
	Undetectable or significantly smaller *I*_K1_	Larger *I*_K1_
Calcium handling	Poor calcium handling	Improved calcium handling
	No or few T-tubules	Abundant T-tubules
Mitochondrial function	Few and underdeveloped mitochondria	Dense, developed, and well-distributed mitochondria
Metabolic characteristics	Glycolysis as major energy source	Fatty acid *β*-oxidation as major energy source

*I*_*K1*_ Inward rectifier potassium, *iPSC-CMs* Induced pluripotent stem cell-derived cardiomyocytes, *MHC* Myosin heavy chain

### Cellular morphology

The iPSC-CMs generated using current protocols are usually round or polygonal with disorganized sarcomeres, whereas adult cardiomyocytes are rod-shaped with an aspect ratio of 5:1–9:1 and organized sarcomeres [101, 102]. iPSC-CMs had lower surface area and volume compared to adult cardiomyocytes [103]. Differences in organelle distribution and morphology between iPSC-CMs and adult cardiomyocytes are also evident. iPSC-CMs in comparison with adult cardiomyocytes lack developed t-tubules networks, which are necessary for efficient contractile function [104]. Furthermore, iPSC-CMs are mostly mononucleated, whereas adult cardiomyocytes are usually multinucleated [105].

### Sarcomeric protein isoforms

During cardiac development, many sarcomeric proteins undergo isoform switching, which can be used as useful markers to determine the maturity level of cardiomyocytes. For example, a switch of the cardiac troponin I (cTnI) isoforms occurs during the maturation of cardiomyocytes. In the fetal heart, expression levels of slow skeletal troponin I (ssTnI) are higher than that of cTnI, whereas cTnI expression levels in adult cardiomyocytes are elevated and ssTnI levels are decreased [106]. Bedada et al. [107] reported that iPSC-CMs mainly expressed ssTnI, indicating their relative immaturity. In addition, myosin heavy chain (MHC) also undergoes isoforms switching during maturation. In human hearts, *β*-MHC (encoded by the MYH7 gene) dominates throughout the development of cardiomyocytes, and levels of which increase with age [108]. Compared with adult cardiomyocytes, the content of *α*-MHC (encoded by the MYH6 gene) in iPSC-CMs is higher. However, *α*-MHC slowly converts to *β*-MHC as iPSC-CMs mature. Finally, fetal cardiomyocytes predominantly express the N2BA isoform of titin, whereas adult cardiomyocytes mainly express the N2B isoform [109]. It was reported that, similar to fetal cardiomyocytes, iPSC-CMs primarily express the N2BA isoform [110]. Therefore, we can judge whether iPSC-CMs are mature based on the ratios of cTnI/ssTnI, *α*-MHC/*β*-MHC, and N2B/N2BA.

### Electrophysiology

Directly comparing differences in electrophysiological properties between iPSC-CMs and adult cardiomyocytes is challenging due to experimental differences, tissue heterogeneity, and disease states. However, the detailed electrophysiological characterization of iPSC-CMs has been reported [111, 112]. Prior studies have documented that iPSC-CMs in comparison with adult cardiomyocytes displayed action potential (AP) phenotypes characterized by smaller maximum diastolic potential and slower upstroke velocity [76, 111]. In addition, it has been reported that the ventricular-like iPSC-CMs shared electrophysiological properties analogous to those of adult cardiomyocytes, including the distinct plateau phase (phase 2) followed by accelerated repolarization (phase 3), the AP duration in the normal range of QT interval, and the maximum diastolic potential which was close to that of adult cardiomyocytes [112].

Different ionic currents have been characterized in iPSC-CMs, including sodium (*I*_Na_), calcium (*I*_Ca_), hyperpolarization-activated pacemaker (*I*_f_), transient outward potassium (*I*_to_), inward rectifier potassium (*I*_K1_), and the rapid and slow activating components of the delayed rectifier potassium currents (*I*_Kr_ and *I*_Ks_, respectively). iPSC-CMs have prominent *I*_Na_ and *I*_Ca_ with activation and inactivation gating properties that are similar to those of human ventricular cardiomyocytes [111, 113, 114]. Ma et al. [111] reported the presence of I_f_ in ventricular-like iPSC-CMs, which promoted auto-depolarization in phase 4. Three K^+^ currents (*I*_to_, *I*_Kr_, and *I*_Ks_) were observed in iPSC-CMs with their maximum current densities and activation characteristics comparable with those of adults CMs [55, 111]. However, *I*_K1_ in iPSC-CMs was either undetectable or significantly smaller than that in adult cardiomyocytes.

In summary, multiple ion channels are present in both iPSC-CMs and adult cardiomyocytes, which lead to characteristic AP. But significant differences do exist, such as reduced *I*_K1_ and the existence of *I*_f_. Consequently, the iPSC-CMs exhibit spontaneous automaticity, which is not recorded in adult cardiomyocytes.

### Excitation contraction coupling and calcium handling

Cardiac contraction and relaxation are accomplished through excitation–contraction coupling, which is the orchestrated cycling of calcium between cytoplasm, sarcoplasmic reticulum (SR), and troponin [115]. The observed whole-cell intracellular Ca^2+^ concentration ([Ca^2+^]_i_) transients in iPSC-CMs demonstrated the presence of excitation–contraction coupling resembling native cardiomyocytes [116]. It has been reported that Ca^2+^ influx into cells through depolarization-activated L-type Ca^2+^ channels leads to the release of SR Ca^2+^ stores via Ca^2+^-sensitive ryanodine receptors, recapitulating a process known as Ca^2+^-induced Ca^2+^ release in iPSC-CMs [117]. It was noted that the Ca^2+^ cycling properties of iPSC-CMs cultured on micro-grooved substrates were significantly improved with a shorter time to peak and more organized SR Ca^2+^ release in response to caffeine [118]. Besides, iPSC-CM-based HCM modeling indicates the existence of functional SR and Ca^2+^ transients [88]. However, Ca^2+^ transients in iPSC-CMs are small with a relatively slow rise characterized by a U-shape waveform, suggesting that the calcium handling properties of iPSC-CMs are relatively immature [119]. Several studies have shown that the Ca^2+^ handling function in iPSC-CMs may be affected by poorly developed SR and T-tubules deficiency [104, 120].

### Mitochondrial function and metabolism

The heart contains a large number of mitochondria, comprising up to 23% in human, 22% in dog, 28% in rat, and 32% in mouse myocardium [121]. The mitochondria of adult cardiomyocytes are mostly rod-shaped and evenly arranged along the sarcomere [122]. Cellular factors controlling mitochondria are highly expressed in adult cardiomyocytes, including fission factor Drp1 and fusion factors Mfn1, Mfn2, and Opa1 [123]. Compared with adult cardiomyocytes, iPSC-CMs have fewer and thinner mitochondria, which usually aggregate near the nucleus with decreased expression of mitochondrial dynamics proteins [120, 124].

During the early stages of cardiac development, cardiomyocytes primarily rely on glycolysis as an energy source (80%). As cardiomyocytes mature and terminally differentiate, mitochondrial oxidative phosphorylation, mainly in the form of glucose oxidation and fatty acid *β*-oxidation, becomes the major energy source (80%). Interestingly, cardiac metabolism in patients with HCM switches back to a more fetal phenotype with an increase in glycolysis and a decrease in fatty acid *β*-oxidation [125]. Similar to fetal cardiomyocytes, iPSC-CMs mainly rely on glycolysis as an energy source [126].

## Approaches to enhance iPSC-CMs maturation

The relatively immature iPSC-CMs make HCM modeling challenging, as it is uncertain whether iPSC-CMs can recapitulate human HCM phenotypes. Furthermore, the understanding of early pathological events would be affected by the maturation levels of iPSC-CMs. Several culture methods and techniques have been proposed to generate mature and homogeneous cardiomyocytes, including prolonged culture time [127], triiodothyronine (T3) hormone treatment [104], microRNA (miRNA) overexpression [128], metabolic manipulation [129], increased substrate stiffness [102], three-dimensional (3D) tissue engineering [130], mechanical stress [131], and electrical stimulation [132]. The general principle of these methods is to simulate in vivo environment and subject iPSC-CMs to relatively stable physical and/or humoral stimuli to promote their structural and functional maturation.

Initial studies tried to promote the maturation of iPSC-CMs by prolonging culture time. For example, late-stage iPSC-CMs (80–120 days of in vitro culture) exhibited increased cell volume, greater density and alignment of myofibril, and a significant increase in the proportion of multinucleated cardiomyocytes [127]. Furthermore, long-term cultured iPSC-CMs showed elevated levels of mitochondrial oxidative phosphorylation, enhanced contractility, and responsiveness to isoproterenol [133]. It has been reported that protein kinase A/proteasome-dependent signaling pathway modulated mitochondrial respiratory chain proteins and enhanced metabolic output of iPSC-CMs during long-term culture, resulting in increased cell contractility [133]. However, the arrangement of sarcomeres in iPSC-CMs remained disordered compared to adult cardiomyocytes, suggesting that additional approaches are required to achieve full maturation.

The use of a T3-containing medium can promote the molecular, morphological, and functional maturation of iPSC-CMs, including increased expression of genes encoding sarcomeric proteins, improved sarcomeric organization, and increased action potential amplitudes and contraction force [134]. Transcriptomic analysis revealed that iPSC-CMs treated with T3 were more mature than those without T3 [135]. In addition, the combination of T3 and glucocorticoids promotes the formation of T-tubules and enhances excitation–contraction coupling [104]. These studies indicate that T3 plays an important role in the maturation of iPSC-CMs. However, Bedada et al. [107] reported that T3 did not affect the cTnI/ssTnI ratio in iPSC-CMs, which could be used as a genetic marker for cardiomyocyte maturity.

Genomics revealed that miRNAs are key regulators during cardiac development [136]. Delivering miRNAs to human embryonic stem cell-derived cardiomyocytes resulted in increased cell size and proportion of binucleated cells, improved sarcomere alignment and calcium handling [137]. Furthermore, overexpression of miRNA in the let-7 family increased cell size and sarcomere length with enhanced contractility and mitochondrial oxidative phosphorylation, promoting the maturation of iPSC-CMs [128, 138].

During cardiac development, the main energy source of cardiomyocytes undergoes a shift from glycolysis to oxidative phosphorylation of fatty acids and glucose [125]. This metabolic change can be mimicked by adjusting medium composition to promote maturation of iPSC-CMs, such as by replacing high-carbohydrate, glucose-based medium with a low-carbohydrate, fatty acid-based medium [129, 139]. However, a subsequent study indicated that culturing iPSC-CMs in a medium with rich fatty acids induced lipotoxicity, causing cell death [140]. To overcome the disadvantage, the medium containing galactose and fatty acids was used to culture iPSC-CMs, which displayed elongated cell morphology, improved sarcomeric organization, increased myofibril force generation, and elevated levels of oxidative metabolism [141].

The dynamic cellular environment of heart tissues cannot be fully recapitulated in monolayer cultured cells. Culture substrates with increased stiffness were thus used to resemble those of the native tissue. It was reported that culturing mouse embryonic stem cells on polymer mattress can promote their differentiation into cardiomyocytes [142]. By screening the library of polymers comprised of polyethylene glycol (PEG), hydrophobic poly-*ε*-caprolactone (PCL), and carboxylated PCL (CPCL), Chun et al. [143] found that culturing iPSC-CMs on a 4% PEG-96% PCL matrix enhanced cell contractility and mitochondrial function, as well as isoform switching from ssTnI to cTnI. Afterward, Feaster et al. [144] cultured iPSC-CMs on a 0.4- to 0.8-mm-thick mattress of undiluted Matrigel (mattress iPSC-CMs) and on a control substrate of 0.1-mm-thick, diluted Matrigel (control iPSC-CMs). Compared with control iPSC-CMs, mattress iPSC-CMs exhibited rod-shaped morphology, increased sarcomere length, and elevated expression levels of cTnI.

Monolayer cell culture models have been widely used in cardiovascular disease research. However, they can neither fully mimic the cellular environment in the heart nor recapitulate the architecture of myocardial tissue. In contrast, 3D cardiac tissues can better simulate the structure and microenvironment of the human heart, which are important for disease modeling [145]. iPSC-CMs can be mixed with scaffolds to form engineered heart tissues (EHTs) [146]. Consisting of collagen, gelatin, hyaluronic acid, and natural extracellular matrix (ECM) extracts, hydrogel scaffolds are frequently used to improve the maturation of iPSC-CMs and serve as a model for measuring myocardial tissues contractility [147, 148].

During human growth and development, subjecting cardiomyocytes to increasing workloads can promote cardiac maturation [149]. Two recent studies demonstrated the feasibility of this approach by modulating tissue stress and electrical stimulation frequency, respectively. Abilez et al. [131] found that increasing the tension of EHTs could promote their maturation, including improved cell alignment and calcium dynamics, and increased expression of mature cardiomyocyte genes. In addition to mechanical stress, electrical stimulation can also improve the maturity of iPSC-CMs. It was reported that iPSC-CMs under electrical stimulation exhibited advanced levels of structural and functional maturity, e.g., adultlike gene expression profiles, remarkably organized sarcomere, the presence of T-tubules, positive force–frequency relationship, and improved calcium handling [150]. Furthermore, the combination of these two approaches was also used to promote the maturation of iPSC-CMs [151].

## HCM phenotypes of iPSC-CMs

With the advancement of culture and differentiation protocols, iPSCs can be efficiently generated and directed to differentiate into cardiomyocytes. Therefore, more and more laboratories use iPSC-CMs to establish in vitro HCM models [89, 152]. However, how do HCM phenotypes of iPSC-CMs compare with those of “real” human HCM? To solve this question, our study statistically analyzed 28 studies reporting phenotypes of human iPSC-CMs either derived from iPSC lines of patients with HCM or from human iPSC lines in which the HCM mutation had been genetically introduced (Table 3).

**Table 3:** Studies reporting phenotypes of human iPSC-CM lines with HCM mutations

Mutation	Cell line	Peak force	T1	T2	Cell size	Disarray	Multinucleation	Other phenotypes	Reference
MYH7 Het p.Arg663His	Patient	NA	NA	NA	+ 60%	+ 250%	50% vs. 20%	Increased normalized contractile motion, elevated [Ca^2+^]_i_, decreased Ca^2+^ release, increased DAD	[79]
MYBPC3 Het Gly999-Gln1004del	Patient	NA	NA	NA	+ 20%	+ 50%	NA	cMyBPC–20%	[153]
MYBPC3 Het Exon 25	Patient	NA	NA	NA	+ 50 − 100%	NA	NA	NA	[154]
MYH7 Het p.Arg442Gly	Patient	NA	NA	NA	+ 15%	+ 150%	NA	Resting [Ca^2+^]_i_ + 20%, decreased Ca^2+^ store, APD prolongation + 60%, arrhythmias + 300%, I_Ca_ and I_Na_ up	[155]
MYBPC3 Het c.2373dupG	Patient	− 50%	NA	NA	±	NA	NA	cMyBPC haploinsufficiency	[156]
TPM1 Het p.Asp175Asn MYBPC3 Het p.Gln1061X	Patient	NA	NA	NA	± (T); + 100% (M)	NA	40% vs. 20% (T); 45% vs. 20% (M)	Prolonged APD, increased arrhythmias (T, not M), increased DAD (M, not T), TPM1 up in T, MYBPC3 up in M	[157]
MYH7 Het p.Glu848Gly	Patient	− 50%	NA	NA	+ 40%	Yes, but not quantified	NA	Increased Ca^2+^ sensitivity, K_Act_ + 60%	[158]
MYH7 Het p.Val698Al	Patient	NA	NA	NA	NA	NA	NA	NA	[159]
MYBPC3 Het c.1358-1359insC	Patient	NA	NA	NA	+ 65%	NA	NA	cMyBPC haploinsufficiency	[160]
TNNT2 Het c.236 T > A	Isogenic	NA	NA	NA	NA	NA	NA	Increased Ca^2+^ sensitivity, decreased peak Ca^2+^ transients, APD50 prolongation + 70%	[161]
MYBPC3 Het Δ25/ p.Asp389Val	Patient	NA	NA	NA	+ 100%	NA	NA	Ca^2+^ transient irregularities + 500%	[162]
MT-RNR2 Het m.2336 T > C	Patient	NA	NA	NA	+ 27%	No	NA	[Ca^2+^]_i_ + 60%, increased SR store, I_Ca_ − 43%, APD prolongation + 14%, increased DAD, mitochondrial 16S rRNA − 55%, MMP − 25%, ATP/ADP ratio − 53%;	[163]
MYL3 Het c.170C > G MYBPC3 Het p.Val321Met	Patient and isogenic	NA	NA	NA	±	No	NA	Increased diastolic [Ca^2+^]_i_, DAD, contraction and relaxation (in MYL3, not MYBPC3) velocity slightly up	[164]
TNNT2 Het p.Ile79Asn	Patient	NA	+ 75%	+ 30%	±	+ 60%	NA	Decreased sarcomere length, increased Ca^2+^ sensitivity, higher Ca2 + buffering, decreased peak Ca^2+^ transients, shortened early repolarization	[165]
MYH7 Het/Hom c.C9123T	Isogenic	− 20% (Het); − 70% (Hom)	+ 20%	±	+ 50%	Yes	2% vs. 1%	Increased DAD, basal and maximal respiration up, increased ATP production, MYH7/MYH6 ratio up	[166]
TPM1 Het p.Asp175Asn MYBPC3 Het p.Gln1061X	Patient	NA	NA	NA	NA	NA	NA	Decreased I_Ca_, increased I_to_ and *I*_K1_, increased DAD and EAD	[167]
MYH7 Het p.Arg723Cys; MYH7 Het p.Arg403Gln; MYH7 Het p.Arg663His; MYBPC3 Het p.Arg943x; TNNI3 Het p.Arg186Gln	Patient	NA	NA	NA	NA	NA	NA	NA	[168]
MYH7 Het p.Glu848Gly	Patient	− 75%	− 30%	− 10%	±	Yes	NA	Impaired fractional shortening, maximal contraction velocity + 15%	[169]
ACTC1 Het p.Glu99Lys	Patient	+ 300% (K1); − 50% (K2)	±(K1); + 50% (K2)	NA	NA	NA	NA	Contraction velocity (K1 up, K2 down), TTP + 115% (K1), CTD90 + 31.4% (K1), increased arrhythmias (K2), increased DAD (K1)	[170]
MYH7 Het p.Arg403Gln; MYBPC3 Het p.Trp792ValfsX41; MYBPC3 Het p.Arg502Trp	Patient	+ 50 − 100%	±	+ 20 − 65%	+ 30%	+ 60%	NA	Maximal contraction velocity + 79%–121%, highly activated p53 and p21 pathways, elevated mitochondrial content, increased ROS, and elevated ratio of ADP/ATP	[171]
MYBPC3 Het p.Arg943x; MYBPC3 Het p.Arg1073Pro	Patient and isogenic	±(Het); − 60% (Hom)	NA	NA	±	±	NA	Increased diastolic [Ca^2+^]_i_, slower Ca^2+^ decay, decreased contraction and relaxation velocity (Hom), downregulated expression of ATP2A2	[172]
ACTN2 Het p.Thr247Met	Patient	+ 19%	±	+ 17%	+ 80%	+ 38%	NA	Increased Ca2 + sensitivity, elevated action potential amplitude, APD prolongation + 50%, increased I_Ca_, transcript levels of ACTN2 + 200%	[173]
MYL2 Het p.Arg58Gln	Patient	NA	NA	+ 90%	+ 29%	+ 100%	NA	Diastolic [Ca^2+^]_i_ − 23%, peak Ca^2+^ transients − 45%, Ca^2+^ decay tau + 38%, I_Ca_ − 45%, increased arrhythmias	[174]
MYH7 Het p.Arg663His; MYBPC3 Het p.Val321Met; MYBPC3 Het p.Val219Leu; TNNT2 Het p.Arg92Trp	Patient and isogenic	NA	NA	NA	NA	NA	NA	Increased diastolic [Ca^2+^]_i_ and Ca2 + sensitivity, slower Ca^2+^ decay, reduced NCX function, decreased sarcomere length, increased maximal contraction velocity, decreased maximal relaxation velocity	[88]
MYH7 Het/Hom c. 9123C > T; ACTC1 Het/Hom c. 301 G > A	Patient and isogenic	− 32 − 53% (M); + 41% (A)	+ 10% (M); + 47% (A)	+ 20% (M); + 38% (A)	Increased	Yes	NA	Increased diastolic [Ca^2+^]_i_ (M, not A), elevated systolic [Ca^2+^]_i_, increased spontaneous beat rate (M, not A), increased arrhythmias, basal and maximal respirations up	[175]
MYH7 Het p.Arg403Gln	Patient	+ 100%	NA	NA	+ 33%	NA	NA	Increased maximal contraction and relaxation velocity	[176]
MYBPC3 Het c.1377delC	Patient	NA	NA	NA	NA	NA	NA	NA	[177]
MYBPC3 Het Δ25/ p.Asp389Val; MYH7 Het p.Arg243Cys	Patient	NA	±	+ 24% (MYBPC3); + 39% (MYH7)	+ 76% (MYBPC3); + 180% (MYH7)	NA	23% vs. 6% (MYBPC3); 54% vs. 6% (MYH7)	Increased Ca^2+^ decay tau, increased arrhythmias, relaxation velocity down	[178]

*ACTC1* Cardiac *α*-actin gene; *AP* Action potential; *APD* Action potential duration; *ATP2A2* ATPase sarcoplasmic/endoplasmic reticulum Ca^2+^ transporting 2 gene; *[Ca*^*2*+^*]*_*i*_ Intracellular calcium concentration; *cMyBPC* Cardiac myosin-binding protein C; *CTD90* Time from peak Ca^2+^ to 90% return to baseline; *DAD* Delayed after depolarization; *Del* Deletion; *Disarray* Abnormal sarcomeric organization; *EAD* Early after depolarization; *Het* Heterozygous; *Hom* Homozygous; *I*_*Ca*_ L-type Ca^2+^ current; *I*_*K1*_ Inward rectifier potassium; *I*_*Na*_ Na + current; *I*_*to*_ Transient outward potassium; *K*_*Act*_* rate* constant reflecting crossbridge turnover rate; *MMP* Mitochondrial membrane potential; *MYBPC3* Cardiac myosin-binding protein C gene; *MYH6/MYH7* *α*-/*β*-myosin heavy chain gene; *MYL2* Myosin light chain 2 gene; *MYL3* Myosin light chain 3 gene; *MT-RNR2* Mitochondrially encoded 16S RNA gene; *NCX* Sodium–calcium exchanger; *ROS* Reactive oxygen species; *SR* Sarcoplasmic reticulum; *T1* time to peak force; *T2* Time from peak to relaxation; *TNNI3* Cardiac troponin I gene; *TNNT2* Cardiac troponin T gene; *TPM* *α*-tropomyosin; *TPM1* *α*-tropomyosin gene; *TTP* Time to peak Ca.^2+^; *Δ25* Δ25 bp intronic deletion in the MYBPC3; ±No difference; *NA* Not assayed

### Cellular morphology

Histologically, HCM features pathological hypertrophy of cardiomyocytes, sarcomere disorder, and myocardial fibrosis. iPSC-CMs harboring HCM mutations exhibited hypertrophied cardiomyocytes and disorganized sarcomere [79, 153, 155]. In addition, previous studies have reported that the proportion of multinucleated cells was significantly increased in iPSC-CMs carrying HCM mutations [157, 166, 178]. However, studies have also shown that iPSC-CMs with HCM mutations varied widely in cell size with surface areas ranging from 800 [156] to > 6000 μm^2^ [178] and volumes ranging from 5.8 [165] to 120 μm^3^ [166]. iPSC-CMs appear extremely low compared to the 15,000–40,000 μm^3^ in adult cardiomyocytes [179]. In addition to culture protocols (e.g., culturing time, medium composition, etc.), differences in imaging techniques and methods of measurement may explain the scatter.

### Altered contractility

Hypercontractility has been reported in several studies of mutations in MYH7, MYBPC3, ACTN2, and ACTC1. The analysis of single-cell video recordings by pixel quantification software confirmed hypercontractility of patient-derived iPSC-CMs with MYH7 R663H mutation [79]. In addition, Cohn et al. [171] measured cardiac microtissues (CMTs) generated from iPSC-CMs with MYBPC3 R502W mutation by traction force microscopy, finding that the diseased CMTs generated increased twitch force and maximum contraction velocity but without changes in contraction time. HCM CMTs exhibited prolonged relaxation half-time, consistent with previously reported that impaired relaxation was the consequence of HCM mutations [15, 180]. Likewise, iPSC-CMs with ACTN2 T247M mutation exhibited increased peak force and prolonged relaxation time, but no change in time to peak contraction [173]. Compared with healthy isogenic controls in which CRISPR/Cas9 was used to correct ACTC1 G301A mutation in patient lines, diseased iPSC-CMs displayed increased cell shortening, prolonged contraction, and relaxation times [175].

However, it has also been reported that iPSC-CMs with MYH7 and MYBPC3 mutations displayed a hypocontractile phenotype. Mosqueira et al. [166] generated a series of isogenic iPSC-CM lines with MYH7 R453C mutation and then formed EHTs to measure contractile properties. Surprisingly, they found that EHTs carrying the HCM mutation exhibited decreased contractile force and prolonged contraction time but little change in relaxation time. Moreover, Seeger et al. [172] used traction force microscopy in MYBPC3 R943x iPSC-CMs treated with dexamethasone, T3, and insulin-like growth factor 1 (IGF1) to characterize their contractility. The diseased iPSC-CMs showed significantly decreased contractile force generation, prolonged contraction, and relaxation kinetics.

The above studies have shown that iPSC-CMs with HCM mutations did not always exhibit hypercontractility, and even mutations in the same genes (such as MYH7 and MYBPC3) might display opposite phenotypes [156, 158, 171, 176]. Furthermore, different mutations may have different pathogenesis at the sarcomere level. For example, haploinsufficiency [160] may be the main pathogenesis of HCM phenotypes in MYBPC3 c.2373dupG iPSC-CMs, whereas activation of the nonsense-mediated mRNA decay (NMD) pathway [172] may be the main cause of HCM phenotypes in iPSC-CMs harboring MYBPC3 R943x mutation.

### Abnormal calcium handling

The myocyte excitation–contraction coupling can be regulated, in part, through modulation of calcium release and uptake [181]. It was reported that L-type calcium channels were excessively activated in HCM iPSC-CMs, resulting in a pronounced increase in Ca^2+^ currents and elevated [Ca^2+^]_i_ [155]. In addition, Lan et al. [79] reported that iPSC-CMs with MYH7 R663H mutation exhibited significantly smaller SR Ca^2+^ release as compared to controls, leading to increased [Ca^2+^]_i_. The increased [Ca^2+^]_i_ can drive the electrogenic Na^+^–Ca^2+^ exchanger (NCX), resulting in further inward flow of Na + and inducing delayed after depolarization (DAD) [175].

The relationship between [Ca^2+^]_i_ and contractile force is a highly regulated biological constant with half-maximal twitch force at [Ca^2+^]_i_ of ~ 0.65 μM [182]. Based on this, Wu et al. [88] defined the ratio of sarcomere contraction rate to Ca^2+^ transient amplitude (d*F*/Δ [Ca^2+^]_i_) as an indicator of myofilament calcium sensitivity, which was significantly increased in HCM iPSC-CMs. In addition, several studies have reported increased Ca^2+^ sensitivity in iPSC-CMs harboring HCM mutations [88, 158, 161, 173]. Clearly, increased Ca^2+^ sensitivity in HCM iPSC-CMs would lead to hypercontractility at lower [Ca^2+^]_i_ and prolonged Ca^2+^ decay time, which is consistent with the clinical phenotypes of preserved systolic function but diastolic dysfunction in HCM patients [165].

### Decreased energetic efficiency

HCM mutations may result in decreased energetic efficiency of the cross-bridge cycle, i.e., inefficient usage of ATP for contraction, and increased oxygen consumption [183, 184]. Likewise, iPSC-CMs with MYH7 c.C9123T mutation showed increased basal and maximal respiration, and elevated ATP production, but decreased contractility [166]. In addition, HCM mutations frequently lead to increased energy depletion in cardiomyocytes. For instance, Toepfer et al. [185] in their study reported that MYH7 mutations increased proportions of myosins in the disordered relaxed state (DRX) conformation and decreased in the super relaxed state (SRX) conformations, which contributed to the significantly decreased phosphocreatine/ATP ratio, and energetic and metabolic stress.

## Prospects of iPSCs in modeling HCM

### Detecting the pathological significance of gene mutations and determining their pathogenicity

A major challenge faced by doctors treating patients with suspected HCM is how to determine the pathogenicity of specific genetic mutations, especially with incomplete gene penetrance and asymptomatic patients. With the development of iPSC technology, the HCM phenotype can be recapitulated in vitro by generating patient-derived iPSC-CMs [173]. Furthermore, HCM mutations can be corrected by gene editing techniques such as the CRISPR-Cas9 system, eliminating differences in (epi)genetic background, and directly comparing the diseased iPSC-CMs with healthy isogenic iPSC-CMs to examine the causality of HCM mutations [88].

### Discovering new disease mechanisms

The iPSC-CMs models can help us discover new cellular mechanisms caused by HCM mutations. For example, Wu et al. [79] proposed that elevated [Ca^2+^]_i_ was a potential mechanism leading to the arrhythmic phenotype in HCM iPSC-CMs, which was validated in a subsequent study. The Jamie lab [175] reported that a combination treatment of dantrolene and ranolazine was performed on the iPSC-CM lines with MYH7 R403Q mutation to decrease [Ca^2+^]_i_. This strategy proved effective in significantly reducing the frequency of arrhythmic events in HCM iPSC-CMs to levels comparable to isogenic healthy controls. It remains to be seen whether this conclusion applies to other HCM mutations.

### Verifying the disease mechanism caused by HCM mutations

iPSC technology allowed us to test the hypothesis generated from human studies. For example, the study of MYBPC3 R943x mutation by Seeger et al. [172] demonstrated that activation of the NMD pathway is the main pathogenesis of HCM. In addition, Prondzynski et al. [160] reported that iPSC-CMs harboring MYBPC3 c.1358-1359insC mutation displayed hypertrophic phenotype and had decreased MYBPC3 mRNA and cMyBPC levels, validating the hypothesis of haploinsufficiency.

### Aiding risk stratification and prognosis

If the cellular phenotype of mutant-bearing iPSC-CMs is stable and reliable, and clinical relevance can be established, the cellular phenotype can serve as an indicator for clinical risk stratification and prognosis. To achieve this goal, in future studies, our efforts should focus on standardizing culture time, optimizing culture conditions, and developing novel maturation methods to generate normalized, mature iPSC-CMs. In addition, long-term, prospective studies of iPSC-CM phenotype/clinical phenotype correlation must be performed in a large number of mutation carriers to determine the natural history and prognosis of HCM.

### Use for gene therapy

Gene editing technologies can specifically repair gene mutations and have broad prospects in the treatment of HCM. Prondzynski et al. [160] applied trans-splicing and gene replacement techniques to increase the expression of MYBPC3 in iPSC-CMs with MYBPC3c.1358-1359insC mutation. Adenovirus was used to, respectively, transfect iPSC-CMs with 5′ and 3′ trans-spliced molecules and MYBPC3 cDNA. However, both 5′ trans-splicing and 3′ trans-splicing strategies were inefficient with the trans-spliced cMyBPC protein not detectable. In contrast, whole-gene replacement increased the expression level of MYBPC3 to more than 80% in comparison with non-transduced control iPSC-CMs and prevented cell hypertrophy [160].

### Testing of existing drugs

A major advantage of the iPSC technology is the ability to test the efficacy of drugs in mutation-specific or patient-specific iPSC-CMs. Several studies have reported that Ca^2+^ channel blocker verapamil can significantly improve HCM phenotypes including myocyte hypertrophy, Ca^2+^ handling abnormalities, and arrhythmia [79, 88]. However, a previous double-blind clinical trial showed that although verapamil improved symptoms in HCM patients, it failed to provide objective clinical benefits, such as exercise capacity [186]. In addition, it was reported that ranolazine ameliorated arrhythmia and reduced the transduction of cellular hypertrophic signaling in iPSC-CMs with MYH7 R453C mutation [166, 170]. However, a randomized, double-blind, phase 2 study showed that ranolazine did not improve exercise capacity, diastolic function, or quality of life in HCM patients [187]. The effects of these drugs at the cellular level did not carry over to the human body, possibly because pathological changes in organs (e.g., myocardial fibrosis) masked the effects of the drugs on cells. Future, more systematic studies will have to determine the validity of this approach.

### Discovery of new drugs

Compared with animal models, the usage of disease- or patient-specific iPSC-CMs can better reflect the possible effects of drugs in humans. With further research, we can obtain more and more iPSC lines carrying different sarcomeric mutations, which can be used for high-throughput screening assays for drug discovery [122]. Efficacy studies of drugs in iPSC-CMs of different genetic backgrounds are actually clinical trials in dishes, thereby reducing the high cost of current drug development and improving efficiency. Moreover, we can optimize patient selection for clinical trials by predicting patient responsiveness to drugs to achieve individualized treatment.

### Testing drug safety

Human iPSC-CMs can be used to screen for drug-induced cardiovascular toxicity in time, including alterations in cardiac cellular contractility, electrophysiology, and viability [188]. For example, breast cancer patient-derived iPSC-CMs treated with doxorubicin showed decreased cell viability, impaired calcium handling, and mitochondrial function, suggesting that breast cancer patients are more susceptible to doxorubicin-induced cardiotoxicity [189]. In addition, iPSC-CMs derived from healthy people also exhibited cardiotoxicity induced by doxorubicin [190]. Therefore, before clinical trials of new drugs, we can test their safety in iPSC-CMs.

## Limitations to iPSC-derived disease models

Although the iPSCs have great therapeutic and translational potential, they show important limitations, such as low reprogramming efficiency, significant differences in the gene expression profiles with embryonic stem cells (ESCs), and teratogenicity.

One limitation is the low reprogramming efficiency of iPSCs, which ranges from 4.4% with modified mRNA and 1% with the retroviruses to as little as 0.001% with the adenovirus and plasmids [191]. By contrast, B cell lines induced by C/EBPalpha can be converted into macrophage-like cells at 100% efficiency within 2–3 days [192]. This shows the fact that the induction of pluripotency by specific factors in contrast to lineage switching faces more barriers, possibly because of the higher degree of epigenetic and transcriptional similarity among mature cells than between mature and pluripotent cell lines [193].

iPSCs were originally thought to resemble ESCs, given their similarity in morphology, proliferation, differentiation potential, and marker expression [48]. However, this similarity was soon doubted, as differences were discovered in the gene expression profiles. It was found that iPSCs retain donor cell-specific transcriptome, along with the DNA methylation signature [194–197]. For example, Marchetto et al. [194] compared the gene expression profiles of iPSCs and ESCs. Their transcriptome analysis showed that iPSCs had insufficient induction of embryonic-specific genes. In addition, a group of genes were upregulated in iPSCs, but they were silenced in ESCs.

The potential teratoma formation after transplantation of iPSCs is another hurdle for clinical application. Incomplete differentiation and difficulty in eliminating undifferentiated cells may lead to potential teratoma formation [198, 199]. However, advances in the culture and differentiation protocol have largely overcome this challenge, resulting in highly pure iPSC-CMs [88, 141]. In addition to the teratogenicity by the undifferentiated cells, differentiated iPSCs still have the intrinsic risk of malignant transformation [200]. The initial use of retroviruses with the integration of viruses into host cell DNA was a contributing factor. Furthermore, the overexpression of c-Myc increases the risk of teratoma formation [47]. To overcome the limitations of retroviral vectors, non-integrating methods have been developed to induce pluripotency, which reduced the risk of genomic integration [201].

Apart from the above limitations, an important limitation to iPSC-derived disease models is present at the drug screening level, considering the fact that it is unlikely to measure all toxicities or side effects merely at the cellular level [202]. Animal studies and clinical human research are required to investigate drug-induced changes and long-term side effects in nontarget tissues. Besides, with the advances in bioengineering technologies, we will be able to perfuse drug solutions on iPSC derivatives (e.g., cardiac, hepatic, pulmonary, and renal cells) to simulate the multiorgan interactions in the human body, as well as to assess toxicities and side effects.

## Conclusions

Since Yamanaka discovered the method of inducing somatic cells into pluripotent stem cells, there has been impressive progress in reprogramming and differentiation protocols. Currently, we can stably acquire a large number of cardiomyocytes from iPSCs, which opens up a new field of HCM research with great potential. Due to the difficulty in obtaining human myocardial tissue and the species differences in animal models, iPSC-CMs have great advantages with their abundant sources. Several studies have demonstrated that iPSC-CM models can facilitate the study of HCM phenotypes and have made important contributions to elucidating molecular mechanisms. In addition, in combination with gene editing technologies such as CRISPR-Cas9 to directly introduce or repair specific HCM mutations, we can directly compare the differences between patient-specific iPSC-CMs and their isogenic control lines to determine the pathogenic role of HCM mutations.

Nonetheless, the research of HCM modeling with iPSC-CMs is still in its infancy. Differences between complex in vivo structures and pathophysiology and simplified in vitro conditions may result in the inability of iPSC-CMs to fully recapitulate the HCM phenotype. Furthermore, one of the challenges is the need to continue to improve the maturity of iPSC-CMs. Previous studies have shown that increasing substrate stiffness [102], mechanical stress [131], electrical stimulation [132], and several other methods can promote the maturation of iPSC-CMs to a certain degree. However, it is unclear how far maturation should improve before iPSC-CMs could be to be generally accepted as a model for studying HCM. Obviously, the use of iPSC-CMs for HCM modeling requires a set of CMs characteristic criteria, including cell morphology, sarcomeric protein isoforms, electrophysiology, calcium handling, mitochondrial function, and metabolism resembling adult cardiomyocytes (as listed in Table 2).

Although the 28 studies reporting iPSC-CMs with HCM mutations vary largely in cell size and no clear consensus is formed in other HCM phenotypes such as contractility, it is too early to conclude that iPSC-CMs cannot provide critical information for HCM models. These differences may reflect the diversity of culture conditions and measurement methods. Therefore, a consensus-implemented approach is needed to standardize the generation and differentiation of iPSCs, normalizing the structure and function properties of iPSC-CMs. We believe that with standardization of culture conditions and culture time, improvement in maturity, and emergence of more comprehensive measurement techniques, more predictive HCM models from iPSC-CMs would be built to better mimic HCM. This will help to provide a better understanding of the pathophysiology of HCM and its individualized treatment.

## Data Availability

Not applicable.

## Abbreviations

3D: Three-dimensional
ACTC1: Cardiac *α*-actin
AP: Action potential
BMPs: Bone morphogenetic proteins
[Ca^2+^]_i_: Intracellular Ca^2+^ concentration
CMTs: Cardiac microtissues
CPCL: Carboxylated poly-*ε*-caprolactone
CRISPR-Cas9: Clustered regularly interspaced short palindromic repeats associated protein
CSRP3: Cysteine- and glycine-rich protein in 3
cTnI: Cardiac troponin I
DAD: Delayed after depolarization
DRX: Disordered relaxed state
DSBs: Double-strand breaks
EB: Embryoid body
ECM: Extracellular matrix
END-2: Visceral endoderm-like
EHTs: Engineered heart tissues
ESCs: Embryonic stem cells
HCM: Hypertrophic cardiomyopathy
HDR: Homology-directed repair
*I*_Ca_: Calcium current
*I*_f_: Hyperpolarization-activated pacemaker current
IGF 1: Insulin-like growth factor 1
*I*_K1_: Inward rectifier potassium current
*I*_Kr_: Rapid activating component of the delayed rectifier potassium current
*I*_Ks_: Slow activating component of the delayed rectifier potassium current
*I*_Na_: Sodium current
iPSCs: Induced pluripotent stem cells
iPSC-CMs: Induced pluripotent stem cells-derived cardiomyocytes
*I*_to_: Transient outward potassium current
MHC: Myosin heavy chain
miRNA: MicroRNA
MYBPC3: Myosin-binding protein C
MYL2: Myosin light chain 2
MYL3: Myosin light chain 3
NCX: Na^+^–Ca^2+^ exchanger
NHEJ: Nonhomologous end joining
NMD: Nonsense-mediated mRNA decay
PCL: Poly-*ε*-caprolactone
PEG: Polyethylene glycol
PTCs: Premature termination codons
SR: Sarcoplasmic reticulum
SRX: Super-relaxed state
ssTnI: Slow skeletal troponin I
T3: Triiodothyronine
TALENs: Transcription activator-like effector nucleases
